# INNOVATING HOME-BASED CARE THROUGH HOME HEALTH DISCHARGE DECISION SUPPORT (HEADS-UP)

**DOI:** 10.1093/geroni/igad104.3748

**Published:** 2023-12-21

**Authors:** Melissa O’Connor, Margaret Brace, Kathryn Bowles

**Affiliations:** M. Louise Fitzpatrick College of Nursing, Villanova University, Villanova, Pennsylvania, United States; M. Louise Fitzpatrick College of Nursing, Villanova University, Villanova, Pennsylvania, United States; University of Pennsylvania, School of Nursing, Pennsylvania, United States

## Abstract

Medicare and private payers rely upon home health (HH) nurses and physicians to evaluate the needs of older adults and decide when ready for discharge. However, there are no national, empirically derived clinical decision support tools to assist clinicians in making these frequent and important discharge decisions. The purpose of this work was to begin development of a clinical decision support tool to support HH clinician decision-making grounded in the evidence and based in real time. Clinician experts, in teams of three: two nurses and one physician reviewed and judged 500 case studies via a secure, dedicated website, making the decision if the older adults should be discharged from services versus recertified for additional care. Our sample matches the national older adult HH population by gender, race and primary diagnosis. The clinician experts recommended over 44.6% of older adults be recertified for more care when in reality, HH clinicians recertified only 22% for additional care. Regardless of insurance status, Blacks were 1.75 times more likely than Whites to be discharged by HH clinicians even though experts recommended more care (p=0.031). Hispanics trended toward being more likely to be discharged by HH clinicians compared to the experts’ recommendation for more care (p=0.094). Our findings indicate that when provided with organized clinical information and ample time to accurately identify the needs of older adults, expert clinicians refer for additional care significantly more often than real-world HH clinicians and that racial disparities exist in current HH discharge decision making.

